# Adherence to strong recommendations of the German Polytrauma Guideline: an analysis of TraumaRegister DGU data over a decade

**DOI:** 10.1038/s41598-026-61248-5

**Published:** 2026-07-15

**Authors:** Katharina Fetz, Dan Bieler, Sebastian Imach, Rolf Lefering, Käthe Goossen

**Affiliations:** 1 Institute for Research in Operative Medicine (IFOM), Witten/Herdecke University, Cologne, Germany; 2 Chair of Research Methodology and Statistics, Witten/Herdecke University, Witten, Germany; 3 University Hospital and Medical Faculty, Heinrich-Heine-University Duesseldorf, Duesseldorf, Germany; 4 Department of Trauma Surgery and Orthopaedics, Reconstructive and Hand Surgery, Burn Medicine, German Armed Forces Central Hospital, Koblenz, Germany; 5 Department of Trauma and Orthopedic Surgery, Cologne-Merheim Medical Center, University Witten/Herdecke, Cologne, Germany

**Keywords:** Trauma, Guideline, Compliance, Adherence, TraumaRegister, Registry, Treatment recommendation, Human factors, Diseases, Health care, Medical research

## Abstract

The treatment of severely injured patients requires complex pre-hospital and in-hospital emergency interventions. The German Guideline on the Treatment of Multiple and/or Severe Injuries (‘German Polytrauma Guideline’) was developed to provide a standardized, evidence-based treatment approach for this purpose. High guideline adherence is supposed to positively affect quality of care and patient outcomes. The aim of this study is to quantify the adherence to the strong recommendations of the German Polytrauma Guideline in a sample of patients treated at all German trauma centres using data from the TraumaRegister DGU (TR-DGU, Trauma Registry) from over a decade (2011–2022). All strong recommendations from the 2016 version of the German Polytrauma Guideline that could be operationalised in the TR-DGU were selected. Adherence to these recommendations was examined for a population of adults (≥ 16 years) with severe injuries (injury severity score ≥ 16) who were directly admitted to a German trauma centre in the period of 2011–2022 and for whom TR-DGU data were available (*N* = 141,073). Adherence rates were calculated at the recommendation level. We found acceptable adherence rates for most of the investigated recommendations in the pre-hospital and in-hospital trauma data combined. Poor adherence rates were observed for pre-hospital decompression of tension pneumothoraxes, pre-hospital administration of tranexamic acid (TXA), exploratory thoracotomy in hemodynamically unstable patients with penetrating injuries, and decompression of space-occupying intracranial injuries. Our results indicate that many of the strong recommendations of the German Polytrauma Guideline are adhered to in the pre-hospital and in-hospital care of severely injured patients in Germany. We have also identified recommendations where measures should be taken to improve adherence. These results need to be interpreted in the context of the limitations of registry-based studies.

The present study is registered as TRDGU project ID 2,023,008 and follows the publication guidelines of the TraumaRegister DGU.

## Introduction

Major trauma is responsible for around 5 million deaths around the world each year^1^. In Germany, approximately 30,000 people experience severe trauma each year^2^. Major trauma refers to severe and often life-threatening injuries sustained by an individual as a result of an accident or traumatic event^3^. These injuries typically involve multiple body systems and organs, necessitating immediate medical attention. Major trauma is most often caused by motor vehicle accidents, falls from significant heights, industrial accidents, assaults, or sports injuries.

Progress in pre-hospital and clinical treatments has significantly reduced the mortality rate of severely injured patients^4–6^. International^7,8^ and national^9,10^ treatment guidelines have been developed to foster evidence-based treatment for severely injured patients. Adherence to trauma guideline recommendations has the potential to reduce mortality after severe injury^11–14^.

Guideline adherence refers to the proportion of patients treated according to a guideline recommendation, which often represents a treatment consensus based on the best available evidence. Previous studies have found that guideline adherence in medicine is generally low^15–17^ and varies widely across centres^17,18^, medical conditions^19^, types of guideline^17,20^, and time period^18^. As a result, many patients do not receive evidence-based care, while others receive unnecessary care that may even be harmful^16^. Guideline adherence studies have the potential to quantify the quality of treatment and identify barriers and facilitators in the implementation of recommendations^13,21^. Adherence to trauma guidelines has previously been investigated by means of surveys^22^ and observational studies at single medical centres^13^. Registry data has been used in the field of urology^23^. In Germany, the treatment of severe trauma patients is prospectively documented by means of the TraumaRegister DGU (TR-DGU), the national registry of the German Trauma Society.

The aim of this study was to quantify the adherence to strong recommendations of the 2016 version of the German Guideline on the Treatment of Patients with Multiple and/or Severe Injuries (‘German Polytrauma Guideline’^24^; in a sample of patients treated in all German trauma centres using TR-DGU data from over a decade (2011–2022).

## Methods

### Study design and data source

This was a retrospective analysis of prospectively collected registry data. The study used TR-DGU (2011–2022) data to investigate adherence to strong recommendations of the 2016 version of the German Polytrauma Guideline.

### Definitions

For the purposes of this study, we define guideline adherence as the extent to which a health intervention is consistent with the corresponding guideline recommendation. As opposed to “guideline conformity” or “guideline compliance”, “guideline adherence” is a term that better acknowledges that there may be legitimate reasons for healthcare practitioners to deviate from recommendations, such as clinical constellations or patient preferences^25^.

For the data interpretation in our current study, we classify adherence rates as follows: >90% as excellent, 80–90% as acceptable, and < 80% as poor. This classification is particularly relevant for recommendations involving interventions where non-adherence may significantly increase mortality.

### German polytrauma guideline

The German Polytrauma Guideline is an interdisciplinary and interprofessional guideline. It was first published in 2002 as a consensus statement, then upgraded to an evidence- and consensus-based guideline in 2011^24^. It is updated at regular intervals, currently every 5 years. Its development follows a rigorous process involving systematic evidence review, formal consensus methods, and multidisciplinary collaboration in a guideline group^26^. It provides detailed, evidence-based recommendations that are graded according to the strength of the underlying evidence and clinical judgments by the guideline group.

Managed and funded by the German Trauma Society (DGU), the 2016 guideline version comprised 307 recommendations covering 38 topic areas^24^. It covered the diagnosis and management of various injuries in the pre-hospital, emergency room, and primary surgical settings. The development process involved representatives from 20 specialist medical societies and additional organizations such as emergency services associations. Guideline development followed the manual of the Association of the Scientific Medical Societies in Germany^26^. Recommendations were developed based on systematic evidence reviews, with teams of content experts proposing wording and strength of recommendations using a three-level scheme for grading:

- Strong Recommendation (GoR A): Measures with this grade of recommendation should be implemented for close to all patients (the wording uses the imperative verb form). They are based on high-quality evidence and strong consensus, and apply to a large majority of patients and contexts.
- Conditional Recommendation (GoR B): Measures with this grade of recommendation should be implemented for the majority of patients (the wording uses “should”). These are supported by moderate evidence or slightly lower consensus, or there are relevant subgroups or contexts where the recommendation does not apply.
- Optional Recommendation (GoR 0): Measures with this grade of recommendation may be implemented (the wording uses “can”). Their evidence base and consensus does not support a clear preference for one alternative intervention over another.

During structured consensus conferences, delegates from all involved medical societies and organizations, referred to as guideline group members, drafted the recommendations. The final consensus wording and strength were determined through a structured consensus process, facilitated by a neutral moderator.

### TraumaRegister DGU

The TraumaRegister DGU (TR-DGU^27^ of the German Trauma Society (Deutsche Gesellschaft für Unfallchirurgie, DGU) was founded in 1993. The aim of this multi-centre database is the pseudonymized and standardized documentation of severely injured patients.

Data are collected prospectively in four consecutive time phases from the site of the accident until discharge from the hospital: (A) pre-hospital phase, (B) emergency room and initial surgery, (C) intensive care unit, and (D) discharge. The documentation includes detailed information on demographics, injury patterns, comorbidities, pre- and in-hospital management, a course on intensive care unit, and relevant laboratory findings including data on transfusion and outcome of each individual. The inclusion criterion is admission to the hospital via the emergency room with subsequent intensive or intermediate care (ICU/ICM) or reaching the hospital with vital signs and dying before admission to the ICU.

The infrastructure for documentation, data management, and data analysis is provided by the AUC - Academy for Trauma Surgery (AUC - Akademie der Unfallchirurgie GmbH) - a company affiliated to the German Trauma Society. The Committee on Emergency Medicine, Intensive Care and Trauma Management (Sektion NIS) of the German Trauma Society provides scientific leadership. Participating hospitals submit their data pseudonymized into a central database via a web-based application. Scientific data analysis is approved according to a peer review procedure laid down in the publication guideline of the TraumaRegister DGU.

The participating hospitals are primarily located in Germany (90%), but a growing number of hospitals from other countries contribute data as well (presently Austria, Belgium, China, Finland, Luxembourg, Slovenia, Switzerland, Netherlands, and the United Arab Emirates). Currently, over 38,000 cases from almost 700 hospitals are entered into the database per year. Participation in the TraumaRegister DGU is voluntary. For hospitals associated with the TraumaNetzwerk DGU (trauma network), however, the entry of at least a basic data set is obligatory for reasons of quality assurance. The present study and protocol was conducted in accordance with the publication guidelines of the TraumaRegister DGU and was registered and approved by the TR-DGU review board as TR-DGU project ID 2023-008.

### Study sample

**Fig. 1:**
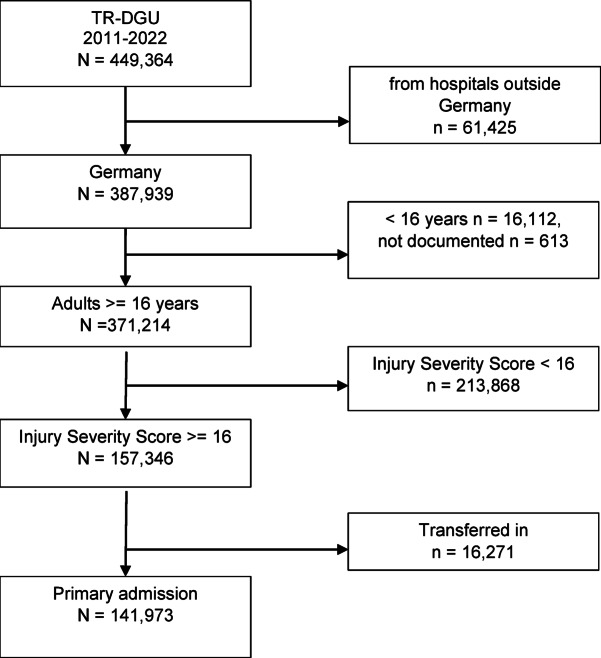
Patient flow with selection criteria.

Data were extracted from the TR-DGU for the years 2011–2022 (*N* = 449,364). Only adult patients (age ≥ 16 years) treated in a German hospital with an Injury Severity Score (ISS) ≥ 16 were included. Patients transferred in from another hospital were excluded. The flow chart in Fig. 1 shows the selection criteria and final sample for our analysis. The final sample most likely matches the target population of the German Polytrauma Guideline.

### Selection of guideline recommendations

Empirical evaluation of guideline adherence requires that both the target populations and the recommended interventions can be operationalised in the registry data with sufficient accuracy. Among the 80 strong guideline recommendations, nine fulfilled these criteria. Recommendation 1.27, which stated that human albumin should not be used for pre-hospital volume therapy, was not included as it was removed in 2022. All other strong recommendations were not included in this study because relevant parameters were not recorded in the registry related to the population (*n* = 23 recommendations) or intervention (*n* = 48). English translations of all included recommendations^28^, along with the corresponding TR-DGU parameters, are shown in Table 1.

### Operationalisation of parameters in the TraumaRegister DGU

The target population of each selected recommendation was operationalised in the TR-DGU, as shown in Table 1. The fraction of the target population that received the intervention (%) is considered the guideline adherence rate. In case the population was difficult to operationalise, or the intervention rate was not exactly measurable, sensitivity analyses were performed using one or more alternative definitions of the target population. The resulting alternative adherence rates were used to test the robustness of the results. For recommendations with more than one possible definition of the target population, our reasoning is explained below in Sect. 2.7.1 to 2.7.4.

In addition, the adherence was calculated for pre-hospital interventions only, and for any early intervention (pre-hospital and/or trauma room). This was done since for some interventions it is important to be performed early, but not necessarily in the pre-hospital setting. Moreover, certain conditions, like pneumothorax, could not exactly be timed using the registry data.

#### Operationalisation of tension pneumothorax

Tension pneumothorax is documented in the TR-DGU via its Abbreviated Injury Score (AIS) code. However, the registry does not record information on the time of diagnosis (pre-hospital or emergency room). As additional criteria indicating an unstable patient a positive shock index > 1, systolic blood pressure < 90 or cardio-pulmonary resuscitation (CPR) have been added to identify patients with a tension pneumothorax needing decompression. The intervention (thorax drainage/needle decompression) was not documented in the reduced basic dataset so that these patients had to be excluded.

#### Operationalisation of massive bleeding

**Fig. 2:**
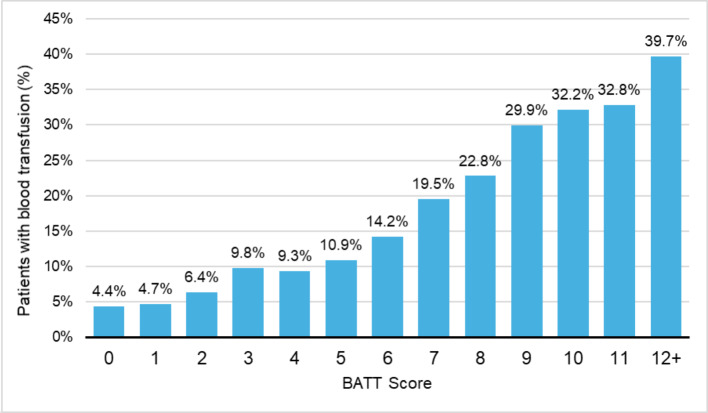
Patients who received packed red blood cells and corresponding Bleeding Audit for Trauma and Triage (BATT) score in our sample.

To assess the recommendation on the use of TXA in massively bleeding patients we had to operationalise massively bleeding patients in the TR-DGU. Massive bleeding is not documented directly in the TR-DGU. In our study, we used the transfusion of packed red blood cells (pRBC) in the trauma room or operation theatre as the primary subgroup. Alternatively, the Bleeding Audit and Triage Trauma (BATT) score^29^ was used to identify patients at risk of massive bleeding. The BATT score is a clinical tool designed to predict the need for massive transfusions in trauma patients. It is based on readily available clinical variables such as blood pressure, heart rate, respiratory rate, presence of penetrating trauma, mechanism of injury, initial haemoglobin levels, FAST results, and base deficit or lactate levels. Validated in a multicentre study, the BATT score demonstrates high accuracy in predicting transfusion needs, enabling early identification and treatment of patients with significant blood loss. Descriptive analyses of our data indicated that the likelihood of pRBC transfusion was 31.6% given a BATT score ≥ 8, and we used this value as a cutoff for our analyses (see Fig. 2).

#### Operationalisation of whole-body computed tomography with a trauma-specific protocol

The TR-DGU contains data for whole-body multi-slice computed tomography (WB-CT) as well as for conventional organ CT. For our study, we included all cases with a WB-CT scan or those with cranial CT plus an additional CT scan of the torso. For further sensitivity analyses we excluded patients who may not have received a WB-CT for clinically valid reasons e.g. patients who died in the first 30 min, or 60 min, as well as patients with penetrating trauma, and isolated traumatic brain injury (TBI).

#### Operationalisation of thoracotomy/penetrating thoracic injury

Acute thoracotomy has been documented in the TR-DGU ever since 2015. Penetrating thoracic injury was operationalised by leading thorax trauma (AIS ≥ 3, all other regions AIS ≤ 2). Hemodynamic instability was operationalised using a mix of criteria including systolic blood pressure ≤ 90 mmHg on admission, or a positive shock index > 1 on admission, or pre-hospital infusion of more than 1000 ml crystalloids, or transfusion of erythrocytes in the emergency room.

### Missing data

Availability of data is generally high in TR-DGU, with rates of missing values < 10% for most variables (Table 2). Missing data were not imputed but patients with missing data were excluded in the specific subgroups. Some parameters, such as hemodynamic instability, were operationalised using several data items. In such cases, patients were included if at least one item was available.

### Statistical analysis

All statistical analyses were performed using SPSS software (version 29, IBM Inc., Armonk NY, USA). Data was analysed descriptively. Frequencies and rates were reported with percentages and numbers of cases, and continuous measures were reported with mean and standard deviation (SD). Guideline adherence was evaluated in the population to which the recommendation applied and calculated as the proportion of patients treated in accordance with the guideline. We used a number of sensitivity analyses to test the robustness of our results to possible operationalisations of the population and/or intervention. Potential reasons for non-adherence, or for a varying degree of adherence over time, were explored by conducting further subgroup and sensitivity analyses.

## Results

We investigated 141,073 patients with severe trauma from 724 hospitals participating in the TR-DGU. The mean age was 55.4 years (SD 21.2), 70.5% were male, the mean ISS was 26.0 (SD 10.7), and in-hospital mortality was 17.8%.

An overview of the strong guideline recommendations, corresponding TR-DGU parameters, and adherence rates is provided in Table 1. Overall, adherence was higher when interventions were assessed across both the pre-hospital and trauma room phases compared with the pre-hospital phase alone.

### Decompression of tension pneumothorax

Adherence to decompression of tension pneumothorax was substantially higher when both the pre-hospital and trauma room phases were considered compared with pre-hospital interventions alone. This pattern remained consistent in the sensitivity analysis restricted to hemodynamically unstable patients with tension pneumothorax.

### TXA application in massively bleeding patients

**Fig. 3:**
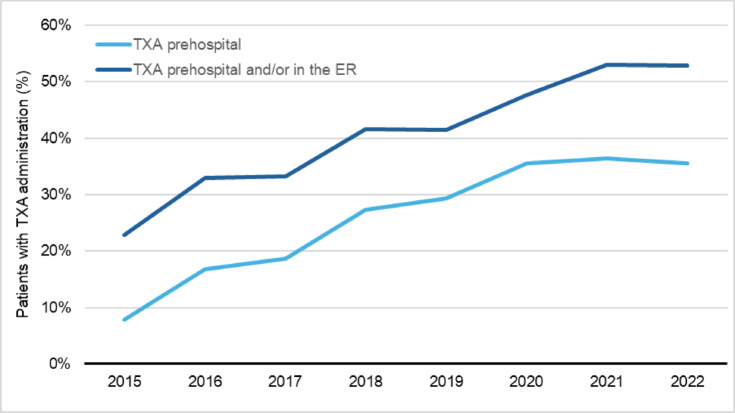
Administration of tranexamic acid (TXA) in patients with BATT ≥ 8 (*n* = 11,693).

For TXA administration, adherence was higher when both the pre-hospital and trauma room phases were considered. Sensitivity analyses using different definitions of massive bleeding showed broadly consistent patterns, with the highest adherence observed in patients receiving pRBC transfusion and substantial pre-hospital volume replacement. Figure 3 shows TXA administration over time in patients with BATT ≥ 8.

### Whole-body CT

WB-CT was performed in the majority of severely injured patients. Lower adherence was observed in clinically distinct subgroups in whom WB-CT may be less feasible or less indicated, including patients who died early after admission, patients with penetrating trauma, and patients with isolated TBI. After exclusion of these subgroups, adherence to the WB-CT recommendation was high.

### Pre-hospital endotracheal intubation in patients with a low respiratory rate

Adherence to pre-hospital endotracheal intubation in patients with a respiratory rate ≤ 6/min was high. However, intubation rates declined over time from 98.2% in 2011 to 92.7% in 2022. Since supraglottic airways have been documented in the TR-DGU since 2015, we additionally considered their use in the later study period. Between 2015 and 2022, supraglottic airways were documented in an additional 5.2% of patients with a respiratory rate ≤ 6/min, resulting in an overall airway-management adherence of 98.1% when both endotracheal intubation and supraglottic airway use were considered.

### Intubation with capnography

Capnography after pre-hospital endotracheal intubation was documented in most cases. Since capnography has only been documented in the TR-DGU since 2015, analyses were restricted to the period from 2015 onwards. Adherence increased over time from 83.9% in 2015 to 90.1% in 2021 and remained high in 2022 at 88.8%.

### Endotracheal intubation in unconscious patients

Adherence to endotracheal intubation in unconscious patients was high and similar whether unconsciousness was defined by pre-hospital GCS ≤ 8 alone or by pre-hospital or admission GCS ≤ 8. Adherence decreased slightly over time from 89.0% in 2011 to 85.1% in 2022. Because supraglottic airways have been documented in the TR-DGU since 2015, we additionally considered their use in the later study period. Including supraglottic airway use increased overall airway-management adherence by 4.9% points, approaching 90%.

**Fig. 4:**
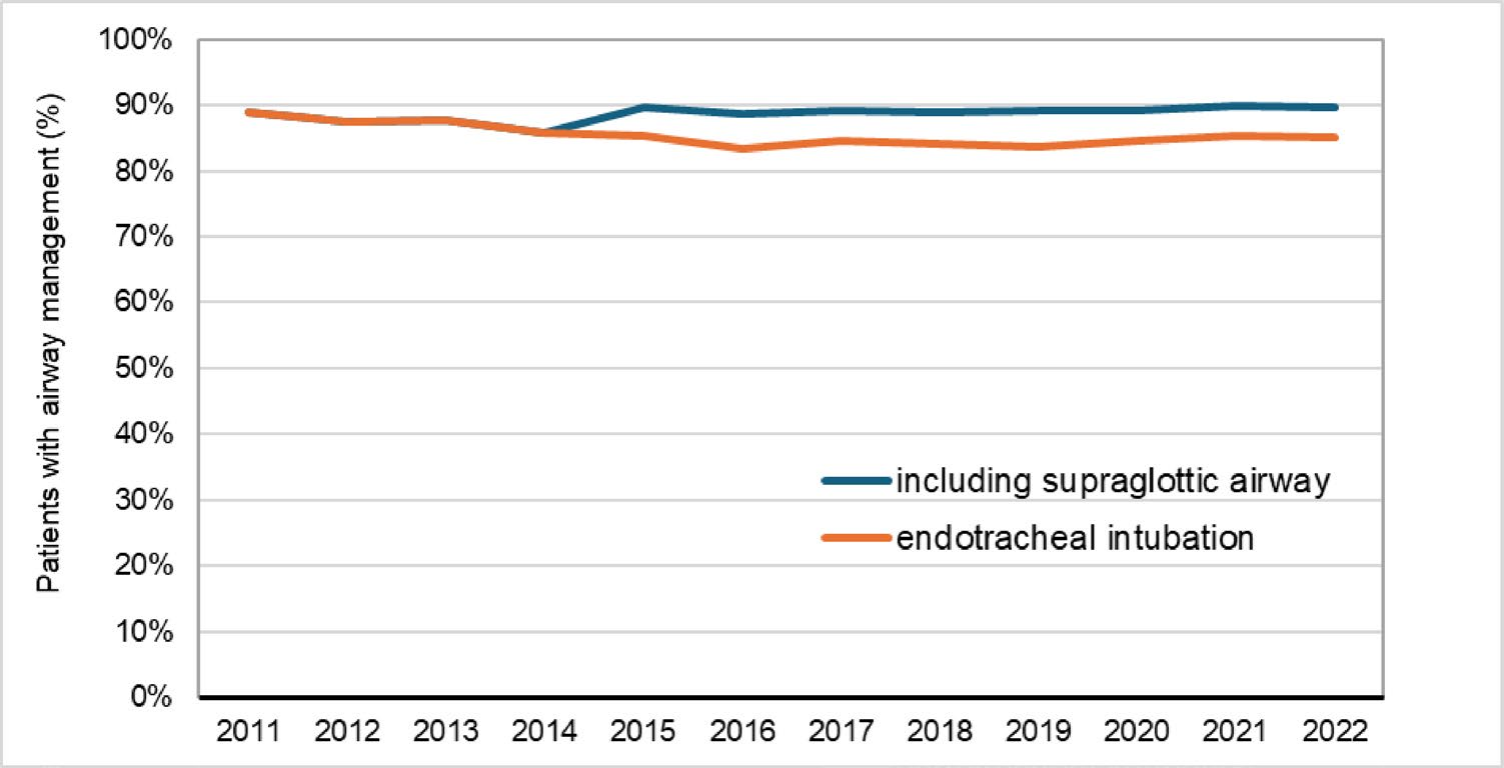
Rate of unconscious patients with airway management per year.

Adherence differed by rescue service type and injury severity. It was higher among patients transported by helicopter than among those transported by ground-based rescue services. When supraglottic airways were included, adherence increased slightly in both groups. Adherence also increased with higher ISS categories, ranging from 78.4% in patients with ISS 16–24 to 92.5% in patients with ISS > 50. Figure 4 shows annual adherence rates for endotracheal intubation alone and in combination with supraglottic airway use.

### Penetrating thoracic injury

Adherence to exploratory thoracotomy in hemodynamically unstable patients with penetrating thoracic injury was moderate. In sensitivity analyses, adherence increased with the number of criteria indicating hemodynamic instability, ranging from 43.2% in patients fulfilling one criterion to 79.4% in patients fulfilling four or more criteria. Adherence also increased with thoracic injury severity, ranging from 26% in patients with AIS 3 thoracic injuries to 70% in patients with AIS 6 injuries.

### Space-occupying intracranial injuries

Data on acute surgical intracranial decompression were available from 2015 onwards. Intracranial injuries were defined by AIS head 4–5, excluding fractures, and TBI diagnosis codes. Patients who died in the trauma room and those without cranial CT were excluded. Surgical procedures performed immediately after trauma room care or on day 0 or 1 after admission were considered urgent.

Adherence to urgent surgical treatment was moderate. It was higher in patients with AIS 5 than AIS 4 head injuries and decreased with increasing age. In patients younger than 60 years, adherence was 55.4%, with an approximate decrease of 10% points per additional decade of age.

## Discussion

This study systematically assessed adherence to strong treatment recommendations of the German Polytrauma Guideline (2016^28^) using data from a large cohort of trauma patients from the TR-DGU of over a decade. Since participation in TR-DGU is mandatory for certified trauma centres in Germany, national coverage has been > 90% since many years, and the included cohort is representative for the severe trauma population in Germany.

There is no general consensus on cutoff values for what constitutes good guideline adherence in the field of acute and emergency medicine. Other authors investigating compliance to trauma guidelines^13,30^ considered ≥ 80% as good compliance, 50 to 79% as inconsistent compliance, and < 50% as poor compliance. For our data interpretation, we classify adherence rates as follows: >90% as excellent, 80–90% as acceptable, and < 80% as poor. This classification is particularly relevant for recommendations involving interventions where non-adherence may significantly increase mortality.

None of the recommendations assessed in our study reached an adherence of 100%, which is in line with earlier research on guideline adherence in this field^13,30^. Overall, we found acceptable adherence rates for most of the investigated recommendations in the pre-hospital and in-hospital trauma data combined. Poor adherence rates were observed for prehospital decompression of pneumothoraxes, pre-hospital administration of TXA, exploratory thoracotomy in hemodynamically unstable patients with penetrating injuries, and decompression of space-occupying intracranial injuries. Each recommendation is discussed in the following paragraphs.

### Tension pneumothorax

For the decompression of tension pneumothoraxes in the pre-hospital setting, we observed poor adherence rates of 27.2% to 38.2%. Adherence rates were higher in the subgroup of unstable patients (SI > 1, SBP > 90 or CPR). Also, adherence rates were higher if preclinical data and in-hospital trauma room data were taken together (81.3% to 85.3%). One major limitation of our data is that we cannot identify the time of tension pneumothorax diagnosis or symptom onset in the TR-DGU. This is because tension pneumothorax is coded as an injury only (AIS 442204.5 according to AIS 2005, Update 2008 Dictionary; Association for the Advancement of Automotive Medicine” (AAAM), USA). It may be possible that some tension pneumothoraxes were not yet clinically manifest in the pre-hospital setting and became symptomatic on the way to the hospital, in the trauma room, or even later.

A possible reason for the low adherence rate of 27% might be that only a minority of pneumothoraxes occurred before hospital admission.

Another possible explanation for the low pre-hospital adherence rate might be training of the emergency doctors. While trauma rooms are staffed by trauma surgeons who are trained and experienced in decompression procedures such as application of chest tubes, pre-hospital Emergency Medical Services (EMS) is often staffed by non-specialist emergency physicians or doctors of other specialties who do not perform such procedures very often. Hence, the low pre-hospital adherence rates may indicate that there is some training need in the detection and treatment of tension pneumothoraxes to reach higher adherence. This aspect is of the utmost importance, since tension pneumothorax is one of the most common reasons for trauma-related preventable death in the pre-hospital setting^31^.

### TXA in massively bleeding patients

For the recommendation for the application of TXA in massively bleeding patients we found poor adherence rates in the pre-hospital setting (29.5% – 49.5%). Adherence rates differed depending on the operationalisation of a massively bleeding patient. Adherence rates increased if data of pre-hospital setting and trauma room were taken together (51.5% − 79.8%). The pre-hospital adherence for TXA administration was below 50% in all subgroups considered, with the highest rates in the subgroup of massive fluid administration and subsequent blood transfusion (49.5%).

Among patients who received TXA pre-hospital, 72% did not require any blood transfusion later in the emergency room, which may be due to earlier pre-hospital administration of TXA.

The low adherence rate to the recommended application of TXA is in line with earlier research: Imach et al. found that there is a significant proportion of patients who did not receive pre-hospital TXA even if indicated^32^. Hamada et al.^22^ found 66% in-hospital adherence rates for TXA application; Rossignol et al.^30^ detected adherence rates of between 27% and 31% of patients, of which only 7% received it in the first three hours after the injury. Also, the median TXA dose was lower than recommended^30^. However, our data only indicates TXA use and provides no further information on dosing or repeated TXA application. The optimal dosage of TXA is currently still the subject of scientific debate^33,34^. In our data, the pre-hospital use of TXA increased in the past eight years from a few cases in 2015 to currently about one third of cases. Our findings on tranexamic acid administration illustrate that changes in clinical practice appear to align more closely with formal guideline implementation than with the publication of individual studies alone, highlighting the central role of guidelines in translating evidence into routine care.

A methodological challenge was to find an appropriate definition of ‘massive bleeding’. It is easy to imagine a massively bleeding patient, but hard to define such patients using registry data like blood pressure, transfusion needs, or administration of large crystalloid volumes. Therefore, in addition to the classical predictors, we used a recently developed and validated instrument to identify patients at high risk for massive bleeding, the Bleeding Audit for Trauma & Triage (BATT) score^29^. Since no general cutoff value for the BATT score is available, we decided to use a BATT score ≥ 8 because blood transfusion rate is above 19% in this population.

### WB-CT

The recommendation to apply a whole-body CT in severe trauma cases is based, among others, on the significant survival advantage found by Huber-Wagner et al. using TR-DGU data^35,36^. We observed comparably high overall adherence rates for the recommendations considering WB-CT (81.8%), especially after exclusion of possible clinically valid reasons for not applying WB-CT (89.2%). Considering low adherence rates in subgroups with high mortality, therapy limitation and consequently, limitation of further diagnostics seems to be a reason for non-adherence here. Also, there was a lower adherence in patients with isolated TBI (cranial CT sufficient) or penetrating trauma (location of injuries is obvious).

### Endotracheal intubation and capnometry/-graphy

The highest adherence rates have been observed in recommendations for endotracheal intubation in selected subgroups (92.0%). Our data indicated a trend of decreasing endotracheal intubation rates from 2011 to 2022. This is a general trend in Germany where pre-hospital intubation has been a rather common intervention in severe trauma in recent decades, compared to other countries^37,38^. This trend, however, has also been shown for patients with GCS ≤ 8 and respiratory rate < 6 where intubation is recommended. When including the use of supraglottic airways, documented since 2015, a higher combined adherence was obtained. Subgroup analyses also showed better adherence for patients with low GCS scores, which is in line with findings from a review investigating guideline adherence in TBI^11^. Furthermore, adherence was higher in Helicopter Emergency Medical Services (HEMS) compared to ground-based EMS, which may be based on a better level of training, or on the limitations during the flight. The effect of knowledge and training on guideline adherence has been reported before in Acute Respiratory Distress Syndrome (ARDS) and use of catecholamines^39,40^.

Capnometry/-graphy is recommended following endotracheal intubation to validate the appropriate position of the tube. Adherence rate in the pre-hospital setting was 86% only but increasing. The current adherence rate is nearly 90%. Capnometry/-graphy is the best-rated quality indicator, based on formal evaluation by experts as well as empirical data from the TR-DGU^41^.

### Penetrating thoracic injury and thoracotomy

This recommendation covers a rather small subgroup of patients (*n* = 350 in 8 years since 2015). We selected only patients with a leading thorax injury who, in addition, had at least one sign of hemodynamic instability. The adherence rate in this group was 50.6% only. However, this rate increased with the number of criteria for hemodynamic instability, or with an increase of injury severity. But a stronger subgroup definition would reduce the even small subgroup to just a handful of cases. For example, all 14 cases with AIS 5 and all four cases meeting criteria for hemodynamic instability received a thoracotomy.

### Space-occupying intracranial injuries

The adherence to the recommendation on urgent surgical intervention for intracranial bleeding was 42.3%. The adherence rate was higher in the more severe cases (55.5% for AIS 5). Since some intracranial bleedings develop over time, they might not have been identified in the initial CT screening. Early surgical treatment decreased in elderly patients, and adherence was 25.3% only in those aged 80 + years.

### Factors influencing guideline adherence

In general, reasons for non-adherence to trauma guidelines may be various. The European Trauma Guideline evaluation study (ETRAUSS-study, Hamada et al.^22^) investigated guideline adherence using physicians’ self-report und found a large variability of adherence (20–96%) and frequent deviations from guideline recommendations. For example, in their study, only 66% reported the use of TXA, only 38% complied with recommended systolic blood pressure targets, and 33% responded that they complied with the target pressure in patients with TBI. Adherence rates differed between European regions and specialties.

The ETRAUSS study found higher guideline adherence rates in anaesthesiologists and intensivists working in trauma centres or trauma ICUs compared to other professions, which may be an indicator of specialist training and routine of practice. Academic affiliation, ICU size and the specialty of trauma team leaders were not linked to better guideline compliance. Other factors influencing guideline adherence may be knowledge levels^13,40^, attitudes, and personal values^22^ Furthermore, other studies reported that feedback and mentorship may be a solution to overcome lack of guideline adherence due to knowledge gaps. Heskestad et al. (2012) observed higher guideline compliance after an educational intervention involving feed-back on performance^42^. Further research has indicated that ICU stay was associated with higher guideline adherence^43^. A factor that might be linked to expertise level of treatment staff.

One study^43^ indicated a positive influence of strength of guideline recommendations on adherence. In our study, we only investigated strong guideline recommendations and cannot provide any findings to compare. Trauma care is a highly stressful and potentially fearful setting where physicians may develop heuristic decision-making strategies to reduce cognitive load^44,45^. This factor may be augmented by lack of knowledge, training and experience, as indicated by studies investigating ARDS^39^. Additionally, higher head injury severity seems to be a factor associated with lower guideline adherence^11,43^, which is in line with our findings regarding application of WB-CT. Therapy limitation in patients with a low likelihood of positive outcomes may not receive full diagnostics anymore. Other research suggests providing shortcut pocket-case summaries of major recommendations to foster guideline adherence^21,46^.

### Implications for research and registries

A major aim of our study was to quantify guideline adherence using a large sample of nationwide trauma registry data. However, we were only able to assess 8 out of 80 strong recommendations of the 2016 version of the German Polytrauma Guideline. The TR-DGU aims to describe the process of care and evaluate the outcomes of severely injured patients. One way to continually evaluate the quality of care is by implementing well-defined quality indicators in the registry and monitoring them over time. Quality indicators are often based on recommendations by experts, or on guidelines^41,47^. However, not all guideline recommendations can currently be mapped to data recorded in the TR-DGU, and their implementation will be considered in future dataset revisions. Our study showed that massive bleeding would be an important additional data item to record in the registry. This would allow more precise evaluation of guideline adherence and assessment of treatment quality. More research is necessary to gain a deeper understanding of underlying mechanisms of adherence and non-adherence in practice. For example, surveys or qualitative interviews with practitioners might contribute to investigate barriers and facilitators considering adherence.

### Implications for training and practice

Overall, our data suggests that guideline adherence rates to recommendations of the German Polytrauma Guideline are acceptable. However, there seems to be some potential for further development. Particularly the training for essential pre-hospital interventions, such as the relief of a tension pneumothorax or the application of capnography, as these are significantly associated with an increase in survival, must be implemented nationwide. The task is not to train all possible pre-hospital interventions in principle, but to focus the training particularly on ensuring that life-saving immediate interventions are used regardless of the profession of the pre-hospital clinical personnel or their specialization. In addition to various course formats, different media such as pocket cards or apps are particularly suitable, ensuring that essential knowledge is easily accessible and always readily available. Furthermore, a feedback structures regarding the quality of care are important for the sustainable implementation of evidence-based practice^15^. This work thus contributes to increasing adherence.

### Limitations

In our study, we were able to investigate the adherence to strong recommendations of the German Polytrauma Guideline (2016) using a large patient sample in a nationwide registry. Nevertheless, our study has some important limitations.

First, we were not able to assess many of the strong recommendations given in the German Polytrauma Guideline. Second, not all populations and interventions are directly documented in the TR-DGU, so that some patient groups (e.g. those with massive bleeding) had to be defined via surrogate parameters. Third, whereas registry data allows us to describe patients who received a recommended treatment, or not, conclusions about possible reasons for non-adherence are not possible based on registry data alone.

The definition of space-occupying intracranial injury was based on the AIS head score as a surrogate parameter, as detailed radiological variables such as midline shift were not available in the registry. This approach may not fully capture the clinical severity of individual cases and may therefore lead to an overestimation of apparent deviations from guideline recommendations.

Alternative approaches, such as surveys and qualitative interviews, may be useful to develop a better understanding of the underlying mechanisms for clinical reasoning and implementation barriers and facilitators.

## Conclusions

- Adherence to many recommendations is good i.e. above 80% of all cases; areas have been identified where measures can be taken to improve implementation.
- Registries are a powerful approach to investigate and quantify trauma guideline adherence.
- Other approaches may be needed to understand underlying mechanisms.

**Table 1:** Strong recommendations (Grade A) from the German S3 Polytrauma Guideline (2016), TR-DGU parameters and pre-hospital and emergency room adherence.

ID and**year**	Strong recommendations from the German Polytrauma Guideline	TR-DGU Parameter	Adherence (% *n*/*N*)	
			pre-hospital	pre-hospital and/or trauma room
1.38 (2011)	Decompress a clinically suspected tension pneumothorax immediately.	*Intervention*: needle decompression or application of a thorax drainage		
		*Patients*:		
		tension pneumothorax (by AIS code)	27.2% (521/1941)	81.3% (1582/1946)
		tension pneumothorax & (SI > 1, SBP < 90 or CPR)	38.2% (266/697)	85.3% (827/969)
2.112 (2016)	In massively bleeding patients, initiate the administration of 1 g tranexamic acid (TXA) over 10 min as early as possible, possibly followed by an infusion of 1 g over 8 h.	*Intervention*: TXA application		
		*Patients*:		
		pRBC transfusion in ER/OP	32.3% (2168/6705)	76.0% (5099/6705)
		pRBC transfusion & BATT ≥ 8	41.0% (1055/2574)	80.2% (2064/2574)
		BATT ≥ 8 only	29.5% (2239/7602)	51.5% (3917/7602)
		pRBC transfusion & pre-hospital volume > 1000 ml	49.5% (1115/2334)	85.0% (1983/2334)
		pRBC transfusion & (SI > 1, SBP < 90 or CPR)	37.7% (1580/4194)	79.8% (3346/4194)
2.126 (2016)	As part of the diagnostic workup for severely injured patients, perform a timely whole-body computed tomography (WB-CT) with trauma-specific protocol.	*Intervention*: WB-CT in the ER		
		*Patients*:		
		all patients	--	81.8% (114,600/140,098)
		Died in the first 30 min		8.1% (130/1610)
		Died in first 60 min		17.8% (506/2844)
		Penetrating trauma		61.2% (3073/5023)
		Isolated TBI		56.3% (13369/23750)
		excluding all of the above		89.2% (98,010/109,894)
1.1 (2011* & 2016)	In polytraumatized patients with apnea or gasping (respiratory rate < 6), perform emergency anesthesia, endotracheal intubation, and ventilation pre-hospitally.	*Intervention*: endotracheal intubation		
		*Patients*:		
		Prehosp. respiratory rate < 6	92.0% (2994/3253)	--
1.11 (2011, mod. 2016)	Use capnometry/-graphy pre-hospitally and in-hospital during endotracheal intubation for tube position confirmation, and afterward for dislocation and ventilation control.	*Intervention*: capnometry		
		*Patients*:		
		Pre-hospital endotracheal intubation	85.9% (14,862/17,311)	-- (capnometry in ER not documented)
2.36 (2011 & 2016)	In unconscious patients (GCS ≤ 8), perform intubation with adequate ventilation (with capnometry and blood gas analysis).	*Intervention*: endotracheal intubation		
		*Patients*:		
		unconscious (GCS ≤ 8) on scene	85.5% (27,702/32,407)	--
		unconscious (GCS ≤ 8) on scene or on admission	85.4% (37,028/43,341)	
3.3 (2011& 2016)	Immediately subject a penetrating thoracic injury causative of the patient’s hemodynamic instability to exploratory thoracotomy.	*Intervention*: acute thoracotomy		
		*Patients*:		
		Leading penetrating thorax injury (AIS ≥ 3 thorax, all other regions AIS ≤ 2) *with* hemodynamic instability (pRBC transfusion; SBP < 90; SI > 1; volume > 1000 mL)	--	50.6% (177/350)
3.23 (2011& 2016)	Treat space-occupying intracranial injuries as a surgical emergency.	*Intervention*: intracranial surgery (immediately after ER or on day 0 and 1 after admission)		
		*Patients*:		
		Brain injury (AIS 4–5), cranial CT performed, survived the ER	--	42.3% (5421/12816)

Abbreviations: AIS = Abbreviated Injury Scale, SI = shock index, SBP = systolic blood pressure, CPR = cardiopulmonary resuscitation, TXA = tranexamic acid, pRBC = packed red blood cells, BATT = Bleeding Audit for Trauma and Triage Score, WB-CT = whole body computed tomography, TBI = traumatic brain injury, GCS = Glasgow Coma Scale, ER = emergency room, OP = operating room.

* some A-Recommendations have been noted already in the earlier 2011^50^version of the S3 polytrauma guideline.

**Table 2:** Availability of selected data in the TR-DGU, based on the whole study cohort of 141,073 cases.

Variable	Rate of missing values
Age	0%
Injuries (AIS)	0%
Hospital mortality	0%
Sex	< 0.1%
Blood transfusion (ER/OP)	0.5%
Whole-body CT	0.7%
Pre-hospital interventions	2.5%
Blunt/penetrating trauma	4.8%
Pre-hospital GCS	6.8%
Blood pressure on admission	7.3%
Pre-hospital heart rate	8.5%
Blood pressure pre-hospital	12.5%
Pre-hospital respiratory rate	34.7%

AIS = Abbreviated Injury Scale, ER = emergency room; OP = operating room, GCS = Glasgow coma scale, CT = computed tomography.

## Data Availability

The data that support the findings of this study are available from TraumaRegister DGU but restrictions apply to the availability of these data, which were used under license for the current study, and so are not publicly available. Data are however available from the authors upon reasonable request and with permission of TraumaRegister DGU.
